# Sudden Cardiac Death and Sudden Cardiac Arrest in Patients with Human Immunodeficiency Virus: A Systematic Review

**DOI:** 10.7759/cureus.13764

**Published:** 2021-03-08

**Authors:** Basel Abdelazeem, Kirolos Gergis, Nischit Baral, Rohit Rauniyar, Govinda Adhikari

**Affiliations:** 1 Internal Medicine, McLaren Health Care, Flint/Michigan State University (MSU), Flint, USA; 2 Internal Medicine, McLaren Health Care, Flint, USA; 3 Internal Medicine, McLaren Flint/Michigan State University (MSU), Flint, USA; 4 Internal Medicine, McLaren Flint, Flint, USA

**Keywords:** sudden death, cardiac arrest, heart failure, systematic review, acquired immune deficiency syndrome (aids), hiv

## Abstract

The importance of this review lies in its study of the risk of sudden cardiac death (SCD) and sudden cardiac arrest (SCA) in people living with the human immunodeficiency virus (PLWH). To the best of our knowledge, this is the first review investigating the effect of the human immunodeficiency virus (HIV) on SCD and SCA.

The review's objective was to determine the risk of SCD and SCA in PLWH. To do this, the electronic databases Ovid MEDLINE, EMBASE, Cochrane Central, Scopus, and Google Scholar were systematically searched to identify eligible studies published before January 31, 2021. Reference lists of the included studies were searched for further identification of relevant studies. The search terms included: "Sudden Cardiac Death," "Sudden Cardiac Arrest," "Human Immunodeficiency virus," "HIV," "Acquired immunodeficiency syndrome," and "AIDS." Only observational studies that assessed the association between SCD and SCA in PWLH were selected.

Data were extracted by two independent authors who screened titles, abstracts, and articles to meet the inclusion criterion. Quality assessment was done by using modified Downs and Black checklist.

A total of seven studies were included in this review. Five studies revealed a higher incidence of SCD in PLWH, two of which focused on patients with HIV and low left ventricular ejection fraction (LVEF). The other two studies were about the association of HIV and SCA. Studies reported that PLWH had a three- to five-fold higher incidence of SCD as compared to non-HIV patients. HIV patients with low LVEF had a higher incidence of SCD than HIV patients with normal LVEF. PLWH had a higher incidence of SCA and less successful cardiopulmonary resuscitation (CPR) as compared to patients without HIV. After adjusting for various confounders in multiple studies, all the studies reported a higher incidence of SCD in PLWH.

To conclude, PLWH is at an increased risk of SCD and SCA. Some risk factors for this include LVEF, viral load (VL), and the cluster of differentiation 4 (CD4) count. There is a paucity of data on the mechanisms involved, although a higher prevalence of cardiac fibrosis and interstitial fibrosis in PLWH may play a role. Because of the general suboptimal quality of the heterogeneous nature of the current evidence, further, rigorous studies are needed to determine the association of increased risk of SCD and SCA in PLWH.

## Introduction and background

Human immunodeficiency virus (HIV) infection was defined by the World Health Organization (WHO, 2007) as positive HIV antibody testing using rapid or laboratory-based enzyme immunoassay and/or positive HIV-ribonucleic acid (RNA) or HIV-deoxyribonucleic acid (DNA) or HIV p24 antigen. The result should be confirmed by a second test [1]. In 2019, 38 million people were living with HIV (PLWH), with around 690,000 deaths from acquired immunodeficiency syndrome (AIDS)-related illnesses worldwide. In 2019, around 2.2 million people were living with HIV in western and central Europe, and North America, with 12,000 HIV-related deaths [2].

Sudden cardiac death (SCD) is a sudden unexpected cessation of cardiac function with hemodynamic collapse, which results in death within one hour of onset. If the patient survived, that event was called sudden cardiac arrest (SCA). In general, most studies reported that the incidence of SCD is 1 or 2 per 100,000 persons per year; however, Couper et al. reported that the incidence is between 0.75 and 11.9 cases per 100,000 persons per year [3]. The underlying etiology and pathogenesis of SCD vary; ventricular fibrillation is the most common cause of SCD. Structural heart disease, including ischemic heart disease, nonischemic cardiomyopathy, and noncardiac conditions like pulmonary embolism, aortic rupture, and other arrhythmias, are also associated with SCD [4]. Few studies reported an association between SCD and HIV. Therefore, in the present study, we performed a systemic review of whether HIV is associated with SCD and SCA, focusing on patients with heart failure.

## Review

Methods

Data Sources and Search Strategy

This systematic review was conducted according to Preferred Reporting for Systematic Review and Meta-Analysis (PRISMA) as recommended by Cochrane Collaboration [5]. A systematic literature review using MEDLINE, EMBASE, Cochrane Central, Scopus, and Google Scholar was performed using the terms "HIV," "AIDS," "Sudden Cardiac Death," and "Sudden Cardiac Arrest" for literature published till January 31, 2021. We included additional articles found in the review of bibliographies or suggested by co-authors based on their relevance to the selected search terms.

Study Selection and Eligibility Criteria

Search results were saved in EndNote files and transferred into Covidence. Two reviewers (BA and NB) independently performed the title and abstract screening to include the articles regarding PLWH, SCD, and SCA. Conflicts were resolved through a third author (KG). Next, we reviewed the selected articles using the following inclusion criteria: randomized controlled trial (RCT); observational study; study population involving adults with HIV; primary outcome SCD, SCA; assessment of incidence and prevalence between HIV and SCD or SCA; English written text; Full text. We excluded case reports and case series with five or fewer cases, studies population children, non-full text articles. Conflicts were resolved through the third author (KG).

Data Extraction

Data from included studies were extracted independently by two reviewers (BA, KG) from Covidence. The consensus was reached in case of any inconsistency with a third author (NB). The data extracted for qualitative synthesis included location, year of study, study design, sample size, population age (in years), and incidence or prevalence of SCD or SCA. The data were entered in Microsoft Office Excel 2016 (Microsoft Corporation, Redmond, WA) by BA and checked by a second author (KG). Any discrepancy was resolved by a discussion between the authors.

Assessment of the Methodological Quality of Included Reviews

We assessed the methodical rigor of the included studies using the modified Downs and Black checklist for RCTs and non-randomized studies [6]. The checklist has 27 items with a total possible score of 28. Papers were rated excellent if they scored above 25, good if they scored between 20 and 25, fair if they scored between 15 and 19, and poor if they scored <15. Each study was assessed by two independent investigators (NB and RR), and discrepancies in scoring were resolved by a third author (BA).

Analysis of Outcome

The primary outcome of interest was the association between SCD and HIV patients with and without heart failure (HF). The secondary outcome was the association between HIV and SCA.

Result

Study Identification and Selection

**Figure 1:**
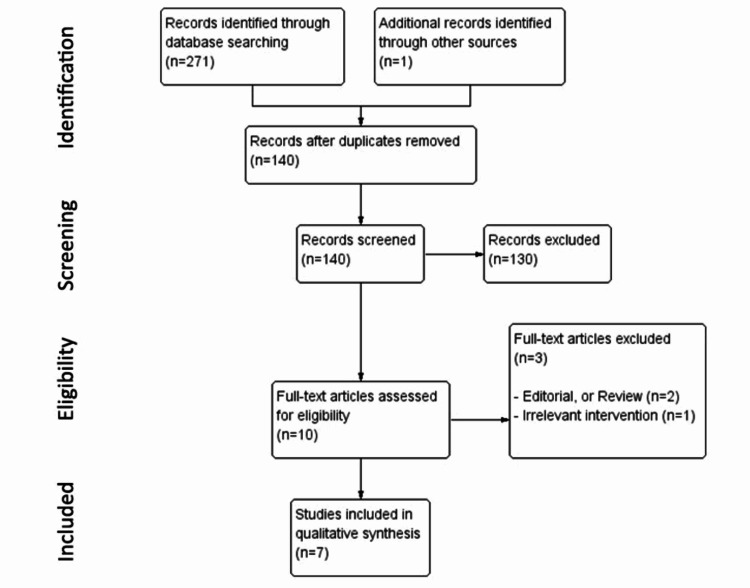
Flow diagram of the study retrieved, excluded, assessed, and included

We identified 272 relevant citations. After removing the duplicates, 140 citations were selected as potentially relevant. The titles and abstracts were screened, and then 10 full-text articles were chosen for further review. Three articles were excluded, one of them was an editorial review and the other two were irrelevant intervention and outcome. Finally, seven articles were included in this systematic review (Figure 1).

Characteristics of Included Studies

The patient's baseline characteristics and summary of the included studies are outlined (Table 1 and Table 2). The patients' baseline characteristics included age, gender, body mass index (BMI), HIV parameters, risk factors, and co-morbidities.

**Table 1:** Baseline characteristics HIV: human immunodeficiency virus; BMI: body mass index; CD: cluster of differentiation; ART: antiretroviral therapy; LVEF: left ventricular ejection fraction; CAD: coronary artery disease; HTN: hypertension; DM: diabetes mellitus; CKD: chronic kidney disease; SIRs: systemic inflammatory response syndrome; NRTI: nucleoside reverse-transcriptase inhibitor; NNRTI: non-nucleoside reverse-transcriptase inhibitor; IQR: interquartile range

Author Year	Group		Age (yrs., mean ± SD)	Gender		Race				BMI (kg/m^2^, mean ± SD)	HIV Parameter					Risk Factors							
				Female	Male	Caucasian	African American	Hispanic	Others		CD4 count	Viral load, copies/ml	ART	Duration of ART prescription (yrs.), median (IQR)	Duration of HIV (yrs.), median (IQR)	Smoking	Cocaine	LVEF (%, mean ± SD)	CAD	HTN	DM	CKD	DLD
Alvi et al 2019 [7].	HIV	344	60±9.7	178 (52%)	166 (48%)		139 (40%)	132 (38%)	73 (21%)	27 ± 5.8	336 ± 244	VL < 200 copies/mL, 221 (64%)	313 (91%)	9 (4–16)	9 (4–16)	162 (47%)	116 (34%)	47±12.0	162 (46%)	221 (64%)	134 (39%)		153 (44%)
	Non-HIV	1805	60±9.4	876 (49%)	929 (51%)		632 (35%)	756 (42%)	414 (23%)	34 ± 5.9						821 (44%)	344 (19%)	48±12.4	581 (32%)	1120 (62%)	628 (34%)		723 (39%)
Yen et al 2019 [8].	HIV	24,306	32.7 +\- 10.0	1504 (6.19%)	22,802 (93.81%)				Taiwanese											887 (3.65%)	557 (2.29%)		576 (2.37%)
	Non-HIV	97,224	32.7 +\- 10.0	6016 (6.19%)	91,208 (93.81%)															4430 (4.56%)	2199 (2.26%)		3490 (3.59%)
Lai et al 2018 [9].	HIV	26272	32.3	1615 (6.1%)	24657 (93.9%)				Taiwanese				19280 (73.4%)					157.44 SIRs (95 % CI) 1.50 (1.31–1.70)	557.13 SIRs (95% CI) 1.11 (1.04–1.19)			495.99 SIRs (95% CI) 1.95 (1.81–2.10)	
Mongarden et al 2015 [10].	HIV	99	44 [38–51]	37 (37.4%)	62 (62.6%)	61%	38%		1% Asian		233 Median [50–489]	42.5 [0–12363]	73 (76%)		9.9 [4.5–15.6]	37			7	15	9		
	Non-HIV	1701	60.1 [50–72.3]	487 (28.6%)	1214 (71.4%)						N/A	N/A	N/A		N/A								
Moyers et al 2014 [11].	HIV and TTE	423	42.3 ± 9.4	80 (19%)	343 (81%)	174 (41%)	147 (35%)	83 (20%)	Asian 14 (3%) Other 5 (1%)	25.0 ± 5.2	274 (89–458)	3.7 (1.9–4.8)	NRTI: 260 (61%) NNRTI: 138 (32%) PI: 229 (54%)			113 (27%)		>50: 339 (80%) 40–50: 423 (9%) 30–39: 29 (7%) <30: 16(4%)	24 (6%)	101 (24%)	38 (9%)	46 (11%)	
Tseng et al 2012 [12].	HIV	2860	39 years (IQR 33-45)	382 (13%)	2478 (87%)	1515 (53%)					353 cells/mm3 (IQR 175-551, mean 390)	The median log viral load was 4.1 copies/mL (IQR 2.9-4.9, mean 3.9)											
Raviglione et al 1988 [13]	HIV	43	37.7 (26-54)	7	36																		
	Non-HIV	293	72 (26-105)	135	158																		

**Table 2:** Literature summary

Author Year	Length of study	Country	Study Design	Study Aim	Participants			Outcome	Summary	Limitation
					Total	HIV	Non- HIV			
Alvi et al 2019 [7].	2011 (19 months)	New York, USA	Retrospective analysis	To determine the incidence of SCD (sudden cardiac death) among PHIV (persons with HIV (human immunodeficiency virus)) with HF (heart failure), who were hospitalized for HF, and the risk factors associated with it	2,578 patients with HF 2,149 (86%) included Excluded 384 pts with ICD and 45 pts unavailable data	344	1805	SCD among HF with HIV	There were 191 SCDs over a median follow-up period of 19 months. Compared to controls, PHIV had a 3-fold increase in SCD [(21 vs. 6.4%), adjusted OR=3.0, CI (1.78—4.24)].	Lack of autopsy - No data on pill counts or prescription refills and adherence to treatment M6 and diet could not be assessed
Yen et al 2019 [8].	2003-2014	Taiwan	Cohort study	To determine SCD risks in Taiwanese patients with and without HIV infection	121,530 patients	24,306 SCD: 97 (0.40) P value <0.001 Incidence per 100,000 person-years: 68.31 P value <0.001 25,061 HIV-infected. Excluded younger than 15 years (n = 33) and those with incomplete data (n = 722)	97,224 SCD: 53 (0.05) P value <0.001 Incidence per 100,000 person-years: 9.31 P value <0.001	SCD in HIV	The study revealed robust associations between HIV infection and incident SCD after stratifying patients according to age, sex, and co-morbidities. HIV infection significantly increased the risk of SCD in all patient subgroups.	The HIV surveillance system did not mandate entering data regarding viral loads and CD4 counts, the indices of advanced-stage HIV infections. - The diagnosis of SCD relied on administrative claims data recorded by physicians or hospitals and was not confirmed by paramedic records, family/witness interviews, or autopsy. - Data of some potential risk factors (e.g., smoking and family history of SCD) were not available for our analysis. - The presence of co-morbidities in the study subjects was determined using the National Health Insurance Research Database and may have been underreported. - External validity of the findings may be a concern because almost all the enrollees were Taiwanese.
Lai et al 2018 [9].	2000 -2014	Taiwan	Retrospective cohort analysis	To determine the incidence of CVD (cardiovascular disease) among PLWH in Taiwan - To examine the effect of active antiretroviral therapy (HAART) on CVD incidence		26,272 The incident density of SCD 54.7, SIRs 3.01 (2.39–3.73) 26355 HIV-infected Excluded younger than 15 years (n = 35) and those with incomplete data (n = 48)		The incidence of CVD among PLWH	Compared with the general population, PLWHA had a higher risk of incident coronary artery disease, percutaneous coronary intervention, coronary artery bypass surgery, sudden cardiac death, heart failure, and chronic kidney disease, but had a lower risk of atrial fibrillation. - HAART reduces risks of incident CVD in PLWHA.	The diagnoses of CVDs that rely on administrative claims data recorded. Maybe less accurate than diagnoses made in a prospective clinical setting. - External validity may be a concern because all the enrollees were Taiwanese.
Mongarden et al 2015 [10].	2000-2012	Paris, France	Retrospective analysis	To determine the epidemiology, characteristics, and outcome, and prognosis of cardiac arrest in HIV- infected patients hospitalized in ICU	1800 cardiac arrest patients	99 cardiac arrest patients	1701 cardiac arrest patients	Investigated causes, clinical features, and outcomes of these patients and assessed the specific burden of HIV on the outcome.	Cardiac arrest (CA) was mostly due to respiratory cause in 36 patients (including 23 cases of pneumonia), cardiac cause in 33 patients (with acute myocardial infarction in 16 patients), neurologic cause in 8 cases, and toxic cause in 5 cases - CA was deemed directly related to HIV infection in 18 cases - In HIV-infected patients, cardiac arrest etiologies are diversified, predominantly cardiorespiratory, and are mostly no related to HIV infection	Retrospective design, including missing data for some details of the medical history - Comparison of patients of several centers with controls of a single center - The study was performed over a long time, in which management has evolved - Authors could not exclude admission bias
Moyers et al 2014 [11].	2000-2009	California, USA	Retrospective cohort analysis	To determine the impact of the left ventricle (LV) on SCD in patients with HIV		2860 consecutive patients with HIV 423 had at least one TTE (transthoracic echocardiogram) 55 AIDS deaths had at least one TTE 13 SCDs had at least one TTE		Effect of LV dysfunction on SCD among HF with HIV	- Lower EF (ejection fraction) had a stepwise and increased hazard of incident SCD but not AIDS deaths. - Hazard of SCD for EF<30% as compared to EF>40% (HR of 22.0, 95% CI 6.0–80.9, p<0.001) -The association between EF<40% and SCD was higher in those with detectable vs. undetectable HIV-RNA (ribonucleic acid) -In conclusion, LV systolic and diastolic dysfunction predict SCD but not AIDS death in patients with HIV, with a more significant effect in those with detectable HIV RNA.	Retrospective data - Only 15% of patients had TTE - Low overall number of SCDS deaths
Tseng et al 2012 [12].	2000-2009	California, USA	Retrospective Cohort analysis	To determine the incidence and clinical characteristics of SCD in patients with HIV		2860 Of the 230 deaths, SCD 30 (13%; 95% CI 9-18%) met criteria for SCDs		Incidence of Sudden Cardiac Death in patients with HIV	SCDs account for a high portion of overall death in HIV patients, and it occurred at a rate more than four times expected. The mean SCD rate was 2.6 per 1,000 person-years (95%CI 1.8-3.8), 4.5-fold higher than expected	Retrospective data collection - Lack of autopsy - Coding practices
Raviglione et al 1988 [13].	1986-1987	New York, USA	Prospective Observational study	To study the outcome of CPR (cardiopulmonary resuscitation) in patients with acquired immunodeficiency syndrome (AIDS)	336 patients underwent CPR	43 with AIDS underwent CPR	293 with diseases other than AIDS underwent CPR	CPR	CPR attempts were markedly less successful in patients with AIDS, despite their younger age, than in patients with other diseases. 1 (2.3%) out of 43 patients with AIDS were revived and survived until discharge compared to 19 (6.5%) patients of 293 with diseases other than AIDS.	Wide range of age groups - Heterogenicity of the patients; some with acute and chronic diseases

Assessments of Bias, Study Quality, and Heterogeneity

The assessment of the methodological quality of the studies ranged from fair to low. Most of the studies included in this systematic review were observational and provided fair quality evidence (Table 3). These studies had a high heterogeneity level due to variability in the participants, interventions, and outcomes reported.

**Table 3:** Modified Down and Black checklist

Author Year	Reporting										Ext. Validity			Int. Validity - Bias							Int. Validity - Confounding						Power	Total Score out of 28	Quality
	No. 1	No. 2	No. 3	No. 4	No. 5	No. 6	No. 7	No. 8	No. 9	No. 10	No. 11	No. 12	No. 13	No. 14	No. 15	No. 16	No. 17	No. 18	No. 19	No. 20	No. 21	No. 22	No. 23	No. 24	No. 25	No. 26	No. 27		
Alvi et al 2019 [7].	1	1	1	0	2	1	1	0	1	1	0	0	1	0	0	1	1	1	1	1	1	1	0	0	0	0	1	19	Fair
Yen et al 2019 [8].	1	1	1	0	2	1	1	0	1	1	0	0	0	0	0	1	1	1	1	1	1	1	0	0	1	0	1	18	Fair
Lai et al 2018 [9].	1	1	1	0	0	1	1	0	1	1	0	0	0	0	0	1	1	1	1	1	1	1	0	0	0	1	1	16	Fair
Mongarden et al 2015 [10].	1	1	1	0	0	1	1	0	1	0	0	0	1	0	0	1	1	1	1	1	0	1	0	0	0	1	1	15	Fair
Moyers et al 2014 [11].	1	1	1	0	1	1	1	0	0	1	0	0	1	0	0	1	1	1	1	1	1	1	0	0	0	0	1	16	Fair
Tseng et al 2012 [12].	1	1	1	0	2	1	1	0	0	1	0	0	1	0	0	1	1	1	1	1	1	1	0	0	0	1	1	18	Fair
Raviglione et al 1988 [13].	1	1	1	1	0	1	0	0	1	0	0	0	1	0	0	1	1	0	1	1	1	1	0	0	0	0	0	13	Poor

Primary Outcome

Our primary outcome of interest was the risk of SCD or SCA in PWLH.

Secondary Outcome

Our secondary outcome was the risk of SCD or SCA in PWLH and having low LVEF and the success rate of CPR in PLWH.

Discussion

SCA leading to SCD is the most common cause of death worldwide, representing more than 50% of all cardiovascular disease deaths [14]. Myocardial fibrosis, primary electrophysiological conditions, and coronary artery disease predispose to SCD. Possible precipitating factors include ischemia, hypoxia, toxins, and electrolyte abnormalities. Combination of susceptible myocardium and precipitating factors lead to ventricular arrhythmias and SCA, followed by SCD if the CPR was unsuccessful in retrieving the patient.

In this systematic review, we included seven studies that reported the effect of HIV on SCD; five studies analyzed the association between SCD and HIV; Yen et al. [8] reported a higher incidence rate of SCD in PLWH than non-HIV patients, 68.31 and 9.31 per 100,000 persons per year, respectively. Lai et al. [9] reported that compared with the general population, PLWH had a higher risk of incident coronary artery disease, percutaneous coronary intervention, coronary artery bypass surgery, SCD, heart failure, and chronic kidney disease but had a lower risk of atrial fibrillation. Tseng et al. [12] reported that SCD account for most cardiac and many non-AIDS natural deaths in PLWH. Alvi et al. [7] and Moyers et al. [11] were mainly on Heart Failure (HF) patients and determined the impact of LV dysfunction on SCD in PLWH. Alvi et al. [7] reported that PLWH had a 3-fold increase in SCD [(21 vs. 6.4%), adjusted OR=3.0, CI (1.78-4.24)]. Moyers et al. [11] reported that the incidence of SCD for EF<30% as compared to EF>40% was significantly higher (HR of 22.0, 95% CI 6.0-80.9, p<0.001).

Tseng et al. [12] involved 2860 HIV patients in the study; out of 230 deaths over 3.7 median years of follow-up, 30 patients met the criteria for SCD (13%; 95% CI 9-18%). SCD accounted for 86% (30/35) of all cardiac deaths. They reported that the mean SCD rate was 2.6 per 1,000 person-years (95%CI 1.8-3.8), 4.5-fold higher than expected. Yen et al. [8] also reported a higher incidence of SCD in HIV patients. The study included 121,530 patients (24,306 PLWH and 97,224 matched controls), from those 5342 (4.40%) of died; among them, 150 (0.12%) died of SCD. Among 150 SCD events, 97 (64.7%) and 53 (33.3%) occurred in PLWH and controls, respectively, which corresponded to incidences of 68.31 in PLWH and 9.31 per 100,000 person-years in controls (P < 0.001). Lai et al. [9] also looked for CVD in HIV patients and reported an incident density of 54.7, Standardized incidence rate (SIR) 3.01 (95% CI 2.39-3.73) for SCD in the subgroup analysis. All three articles agreed that the PLWH has a higher incidence of SCD than the general population, and it was statistically significant.

Both Alvi et al. [7] and Moyers et al. [11] were interested in the HIV patient who had HF or LV dysfunction. Alvie et al. [7] studied 2,578 HF hospitalized patients; 344 were PLWH. And they subclassified the patient according to the LVEF <35%, 35-49%, >50%. 191 SCDs occurred over 19 months of the follow-up period and reported that the rate of SCD was higher among PLWH compared to non-HIV infected individuals (21 vs. 6.4%, p<0.001) [adjusted OR=3.0, CI = (1.78-4.24)]. They also reported that low LVEF, low CD4, and higher viral load were predictors of SCD. Moyer et al. [11] determined the impact of LVEF on SCD in PLWH. They included 423 patients who had at least one transthoracic echocardiogram, and the results revealed that the lower the EF, the higher the risk of SCD. The risk of SCD for EF<30% was higher compared to EF>40% (HR of 22.0, 95% CI 6.0-80.9, p<0.001). Both studies showed that HIV patients with HF have a higher rate of SCD than HIV patients without HF, and the lower the EF, the higher the SCD events.

Two studies analyzed the association between SCA and HIV, Mongaeden et al. [10] described the burden of HIV on cardiac arrest by comparing 99 HIV to 1701 non-HIV patients with a primary outcome of determining CA characteristics in HIV patients in ICU from 2000-2012 in France. They reported that out of the 99 HIV patients, 33 patients sustained cardiac arrest, out of which 18 were directly related to HIV infection. Raviglione et al. [13] studied the association between CPR and HIV. They compared 43 HIV patients to 293 Non-HIV, with the primary outcome of the success of CPR. The results revealed that CPR attempts were markedly less successful in a patient with HIV.

This study has many limitations. First, the quality of included studies was limited to fair, with one poor article. Variation in study quality contributed to the heterogeneity of findings noted. Other sources of heterogeneity are likely to include population differences, including age as a factor contributing to SCD or SCA. Second, all of the included studies were observational studies (prospective or retrospective) affecting the extracted data. Some medical history details can be missed, which leads to selection and information biases. Third, all of the articles lacked the autopsy findings for the SCD cases, so all cases of SCD were presumed not confirmed. Usually, a histopathological exam of the heart is essential to know the underlying etiology [15]. Fourth, Sudden death in PLWH with CD4+ T cell count < 50/cm has been termed as HIV death rather than SCD. The cause of death in such circumstances is severe immunodeficiency rather than SCD. Alvi et al. [7], Mongarden et al. [10], Moyers et al. [11], and Tseng et al. [12] reported that their patients had a CD4+ T cell count of more than 50/cm. But Yen et al. [8], Lai et al. [9], and Raviglione et al. [13] didn’t report CD4+ T cell count for their patients. Fifth, external validation was questionable in all of these studies, as these studies were in a specific location and lacked randomization.

To the best of our knowledge, this is the first review investigating the effect of HIV on SCD and SCA.

## Conclusions

This systematic review revealed a higher rate of SCD and SCA in a patient with HIV than in the general population. The risk is even higher in HIV patients with low LVEF. After adjusting for various confounders like age, sex, and race, the risk factors for cardiovascular disease, persistently low CD4 counts, and high viral loads are also associated with increased risk of SCA and SCD in PLWH. There is a paucity of data on the mechanisms involved, although the prevalence of cardiac fibrosis and interstitial fibrosis in PLWH may play a role. Because of the general suboptimal quality of the heterogeneous nature of the current evidence, further rigorous studies are needed to determine the association of increased risk of SCD and SCA in PLWH.
